# Is it Possible to Reuse the University of Pennsylvania Smell Identification Test (UPSIT ^®^ )? The “2 Zs” Protocol

**DOI:** 10.1055/s-0045-1810003

**Published:** 2025-09-26

**Authors:** Lissa Okamura Asada, Jaqueline dos Santos Andrade, Lisandra Cardoso Bueno, Richard Louis Voegels, Fábio de Rezende Pinna, Marco Aurélio Fornazieri

**Affiliations:** 1 Department of Surgery, Universidade Estadual de Londrina (UEL), Londrina, PR, Brazil; 2 Department of Otorhinolaryngology, Universidade de São Paulo, São Paulo, SP, Brazil; 3 Department of Medicine, Pontifícia Universidade Católica do Paraná (Londrina Campus), São Paulo, SP, Brazil; 4 Department of Otorhinolaryngology: Head and Neck Surgery, Smell and Taste Center, Perelman School of Medicine, University of Pennsylvania, Pennsylvania, United States

**Keywords:** smell, olfactory perception, olfaction disorders, anosmia, economics

## Abstract

**Introduction:**

The University of Pennsylvania Smell Identification Test (UPSIT ^®^ ) is a reliable olfactory test that is easy and quick to apply. It is a one-time use test and its reuse is not recommended by the manufacturer.

**Objectives:**

Because in high-demand healthcare facilities testing all patients with UPSIT ^®^ can be costly, in this present study we sought to assess the feasibility of reusing the UPSIT.

**Methods:**

Two hundred and ninety healthy volunteers aged 18 and 40 without olfactory complaints participated in the study. Two hundred and forty volunteers underwent the UPSIT ^®^ scratching the small strip of the test doing only 2 Z-shaped scratches (“2 Zs” protocol); each UPSIT ^®^ was used 20 times by same-sex participants. The remaining fifty patients performed the UPSIT ^®^ in the standard single-use fashion. Adjusted multiple regressions relating to the olfactory test score, number of times the test was applied, and demographic variables were performed.

**Results:**

The mean scores using the UPSIT ^®^ repeatedly were lower than those done in the single-use standard fashion. Despite this, the scores remained stable until the tenth time the test was applied. When patients perform the UPSIT ^®^ in the “2 Zs” protocol, a correction factor of more 3 points was required in the final score.

**Conclusion:**

UPSIT ^®^ reuse up to 10 times is feasible when a 3-point correction factor is added to the final score. The “2 Zs” protocol described is an alternative to reduce costs in epidemiological studies. This study, however, is limited as it was carried out only with healthy individuals.

## Introduction

Anosmia affects about 5% of the world's population, and hyposmia affects 15 to 25%, which indicates a high and significant prevalence of this disorder. 1 This prevalence increases with age, affecting 14 to 22% of patients over 60. 2 Anosmia impairs the quality of life concerning social and mental anxiety, eating and weight disorders, and depression, as well as neurodegenerative diseases. 3 Despite this impact, patients are often unaware of their olfactory disorder or cannot estimate the severity of their hyposmia. Most physicians are also not prepared to diagnose olfaction complaints. 3

An objective olfactory test is necessary for the diagnosis of olfactory loss, given the low reliability of the patient's subjective assessment. 4 5 The University of Pennsylvania Smell Identification Test (UPSIT ^®^ ) is an objective psychometric and practical test capable of estimating the loss of smell through a questionnaire with four booklets with ten odors, each containing one odor per page. 6 The test is self-administered and has high short- and long-term test-retest reliability. 7 However, the use of UPSIT ^®^ in Brazil is still very recent and faces expensive costs. 8 In principle, the test can only be used once, and the reuse of the exam booklets would reduce considerably the costs. There are no studies that tried to reuse UPSIT ^®^ , and the present study seeks to determine the feasibility of reusing it.

## Methods

### Population

Two hundred and ninety volunteers aged 18 to 40 years who did not have olfactory or taste complaints and did not have upper respiratory tract infections on the day of the visit were included. Regarding exclusion criteria, participants with Parkinson's disease, Alzheimer's disease, epilepsy, stroke, brain tumors, limb paralysis, severe traumatic brain injury, smoking, current nasal disease, and comorbidities were excluded from the study. An Informed Consent Term was presented and signed by every study participant. The project was approved by the Research Ethics Committee of the State University of Londrina under approval number 1,073,331.

### Design of the Study

To test the reliability of using UPSIT ^®^ repeatedly, twelve tests were used 20 times, each time by a different volunteer, totaling 240 volunteers. Six tests were used for men and 6 for women. Each volunteer underwent the test only once. These tests were performed according to the “2 Zs” protocol described below. The results were compared with the remaining 50 participants, matched by sex and age for participants who took the reused test, did the UPSIT ^®^ in the standard single-use form, scratching the entire strip with the microcapsules on each test page. 7 All participants received a normal olfactory test and no olfactory tests, ≥ 35 for women and ≥32 for men (UPSIT). 8

### The “2 Zs” Protocol, a Form to Reuse the UPSIT ^®^

**Fig. 1:**
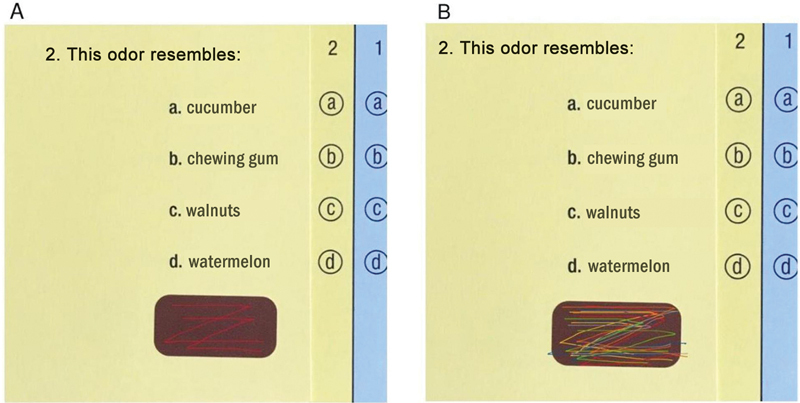
Illustrative images of the University of Pennsylvania Smell Identification Test (UPSIT ^®^ ) reuse form with 2 scratches (“2 Zs” protocol, A) and UPSIT ^®^ reused 10 times in this form (B).

The UPSIT ^®^ consists of four booklets of 10 odors, with one smell per page. The stimuli are embedded in plastic microcapsules in a brown strip at the bottom of each page. Participants scrape off the brown strip using a pencil and scraping the odor is released through. After smelling the odor, the patient must answer a multiple-choice question that describes the odor. The test score is the number of correct answers, which classifies the olfactory function of the individual in normosmia, microsmia, and anosmia. 8 In the “2 Zs” protocol, instead of scraping off the entire brown strip at the bottom of each page, the participant was instructed to make a maximum of 2 Z-shaped scratches. Then, he brought the booklet 1 cm closer to his nose, felt the released smell, and obligatorily marked the answer on a separate sheet. He was advised to make the scratches outside the already scratched part by a previous volunteer ( Fig. 1 ). As we were still in the period of the COVID-19 pandemic, for health precautions, we did not use the same test with an interval of four days. 9

### Statistical Analysis

UPSIT ^®^ scores were described as means and standard deviations. The Shapiro-Wilk test was used to verify the distribution of the samples. The non-parametric Kruskal Wallis and Dunn's *post hoc* tests were done comparing the values of the UPSIT ^®^ among the different number of times it was used. Multiple linear regression was performed to verify the interference of sex, education, and age on the UPSIT ^®^ scores made in the “2 Zs” protocol. The significance level used was 0.05. The statistical software used was STATA 17 (StataCorp LP, College Station, TX).

## Results

**Fig. 2:**
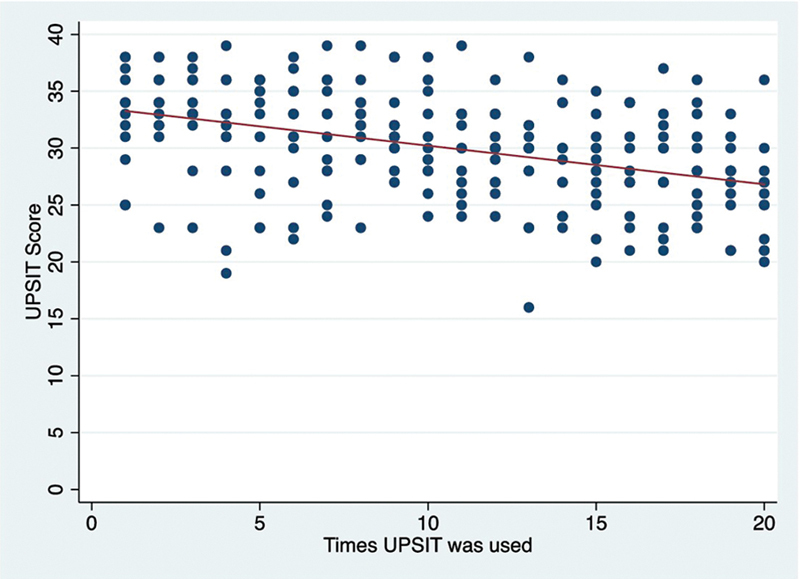
The University of Pennsylvania Smell Identification Test (UPSIT ^®^ ) scores decrease the more times test booklets are reused in the “2 Zs”modality (n = 240, Spearman's rho = -0.43, p < 0.001).

Demographic characteristics of patients can be seen in Table 1 . Table 2 and Fig. 2 show UPSIT ^®^ means according to the test application times. When used repeatedly the scores were lower than when performed in the standard single-use standard form [mean ± SD: 30 ± 4.7 vs. 34.5 ± 3.3, p < 0.01]. There was no statistical difference between the test means when used from one to ten times in the “2 Zs” modality (ps > 0.05, Supplementary Table S1 ).

**Table 1:** Sociodemographic characteristics of the participants

Variables	Total N = 290
**Age, mean (SD), y**	27.3 (7.2)
**Sex, No. (%)**	
Male	145 (50)
Female	145 (50)
**Educational Level, No. (%)**	
< Middle School	6 (1.7)
Middle School	1 (0.4)
Some High School	33 (11.7)
High School	110 (38.3)
Some College	90 (30.8)
Bachelors or Higher	50 (17.1)
**Race, No. (%)**	
Non-White Brazilians	75 (25.8)
White Brazilians	215 (74.2)

**Table 2:** Means and standard deviations of the University of Pennsylvania Smell Identification Test (UPSIT ^®^ ) scores according to the times the test was used using the 2 “Zs” technique

Times used	UPSIT ^®^ score Mean (SD)	Times used	UPSIT ^®^ score Mean (SD)
**1**	**32.7 (4.5)**	**11**	**30 (4.3)**
**2**	**33.4 (4)**	**12**	**29.7 (3.4)**
**3**	**33 (4.1)**	**13**	**28.4 (5.6)**
**4**	**30.9 (5.8)**	**14**	**28.5 (4.4)**
**5**	**31.3 (5.1)**	**15**	**28 (4.3)**
**6**	**31.3 (5.1)**	**16**	**28.3 (4.4)**
**7**	**31.4 (4.7)**	**17**	**28.3 (4.8)**
**8**	**32.1 (4)**	**18**	**28.4 (4.4)**
**9**	**31.8 (3.4)**	**19**	**27.1 (3.6)**
**10**	**30.6 (4.2)**	**20**	**25.7 (4.5)**

Twelve tests were used, therefore the mean represents the scores of 12 times the UPSIT ^®^ was done for the first time, 12 times it was done for the second time, and so on. The mean of 50 tests used in the standard fashion by fifty sex and age-matched participants was 34.5 (SD: 3.3).

A correction factor was calculated based on the stable scores between the first and the tenth time the UPSIT ^®^ was used in the “2 Zs” protocol. The calculation was done by decreasing the UPSIT ^®^ punctuation mean when done in the single-use standard fashion and the average of the means from the first to the tenth time the test was performed in the repeated modality [34.5 - ((32.7 + 33.4 + 33 + 30.9 + 31.3 + 31.3 + 31.4 + 32.1 + 31.8 + 30.6)/10)]. The difference obtained was 2.65, and we rounded it up to an additional 3 points that need to be added to the final score when the UPSIT ^®^ is done in the repeated protocol. Based on the significant drop in scores, mainly after the eleventh time the test was used, we do not recommend reuse after the tenth time.

To verify whether other variables interfered with the UPSIT ^®^ score when performed repeatedly, we set a multiple linear regression model, as follows: UPSIT ^®^ score = times UPSIT ^®^ was used + sex + age + race+ schooling. The more times the test is applied and being a man negatively interfered in the final score. The model coefficients can be seen in Table 3 .

**Table 3:** Multiple linear models showing the effect of the times UPSIT ^®^ was used, sex, race, and schooling on the UPSIT ^®^ score

Variables	ME	95% CI	p value
**Times UPSIT was used**	**-0.31**	**-0.41- -0.22**	**<0.001**
**Male sex**	**-2.59**	**-3.6- -1.6**	**<0.001**
Age	0.01	-0.07- 0.08	0.85
White	-0.49	-1.73- 0.75	0.44
Schooling	-0.84	-1.39- -0.3	0.11

Values less than 0 indicate a negative effect, whereas those above 0 are positive. ME = marginal effect. Schooling= 0 (< middle school) to 5 (bachelors or higher). Rows in bold are statistically significant measures.

## Discussion

The UPSIT ^®^ is an olfactory test spread worldwide and validated for different cultures. It contains forty odors and is highly reliable and reproducible, classifying patients' olfactory dysfunction into up to six categories known as normosmia, mild, moderate, and severe microsmia, anosmia, and simulators. This study makes it possible to use the UPSIT ^®^ more than once, which may reduce the cost of this high-quality olfactory test. We show that using the described “2 Zs” protocol and adding three points to the final UPSIT score it is possible to use this test up to ten times.

As seen in the results section, the single use of this test gives an average score of 3 points higher than the repeated method up to 10 times of reuse. However, this difference can be solved by adding the correction factor of three points to the final score. Other areas use these post-analytical correction factors. 10 The feasibility of reusing olfactory tests is essential to reduce costs and facilitate access to a reliable analysis of the olfactory function. An example is the applicability of the Connecticut Chemosensory Clinical Research Center Test (CCCRC), a low-cost and easy-to-apply test that can be reused within 90 days without interfering with final score. 11 The main disadvantage of CCCRC is the impossibility of its self-application. The UPSIT ^®^ is self-applied, and its application is faster (average of 20 minutes). 6 8 Proper reuse of the UPSIT ^®^ makes it possible to expand its use beyond specialized outpatient clinics in otorhinolaryngology since olfactory disorders precede and accompany several medical conditions in several areas, such as neurodegenerative diseases. 12 13 14 15

Sanitation can be a concern when tests are used repeatedly, particularly after the COVID-19 pandemic period. However, it is worth noting that many smell tests, such as the Sniffin' Sticks and the CCCRC, are for multiple uses. An interval of four days is advisable to reuse the UPSIT ^®^ , bearing in mind that SARS-CoV-2, for example, can survive up to 72 hours on some surfaces. 9 16

As expected, the multiple linear regression model showed that the more times the UPSIT ^®^ is used, the worse the score and that women also outperform men in this repeated modality. It is known that women have better olfactory acuity compared to men. 17 Race, education, and age did not interfere in the final score, which can be explained by the slight difference usually found between the olfactory function between whites and non-whites, the few participants with low education, and the relatively short age range of our sample. 18

Among the strengths of this study are the controlled study design, the large sample size, and the description of how to reduce costs using a gold-standard olfactory test. Our limitations are the convenience sample and data collection in only one recruitment center, which may impair our findings' generalization. The study's limitation lies in its exclusive focus on individuals without prior olfactory disorders. Therefore, the applicability of our findings to those with microsmia or previous anosmia remains uncertain. Generalizing our results to a broader population with pre-existing olfactory conditions should be approached with caution. Future research involving diverse olfactory profiles is warranted to enhance the robustness of our conclusions.

## Conclusion

This is the first study demonstrating that UPSIT can be reused up to ten times when adding 3 points to the final score. Repeated modality of this type of scratch-and-sniff olfactory test can considerably decrease the costs of testing patients' sense of smell with a high-standard olfactory test. These findings can help make the olfactory assessment a universal reality for screening and evaluating patients with olfactory disorders, such as performing audiometry for subjects with hearing loss. It is worth mentioning here that our study was carried out only with individuals without previous olfactory complaints. Therefore, more studies involving people with olfactory disorders will be necessary to increase the robustness of our conclusion.
