# Supplementary material for: Is it Possible to Reuse the University of Pennsylvania Smell Identification Test (UPSIT ® )? The “2 Zs” Protocol
Source: Int Arch Otorhinolaryngol. 2025 Sep 26;29(3):1–5. doi: 10.1055/s-0045-1810003 (PMC12473524; doi:10.1055/s-0045-1810003)
Supplement: Supplementary file 1 — Supplementary Material [file 10-1055-s-0045-1810003-s231570.pdf]

Supplementary Table S1 Dunn pairwise comparison of the University of Pennsylvania Smell Identification Test (UPSIT®) score by the number of times the test was used

	1	2	3	4	5	6	7	8	9	10	11	12	13	14	15	16	17	18	19
2	-0.50																		
3	-0.26	0.23																	
4	0.74	1.24	1.00																
5	0.59	1.09	0.86	-0.14															
6	0.70	1.20	0.97	-0.03	0.11														
7	0.70	1.20	0.97	-0.03	0.11	0.00													
8	0.28	0.78	0.55	-0.45	-0.30	-0.42	-0.41												
9	0.57	1.07	0.83	-0.17	-0.02	-0.13	-0.13	0.28											
10	1.21	1.71*	1.48	0.47	0.62	0.50	0.51	0.93	0.64										
11	1.53	2.03*	1.80*	0.79	0.94	0.83	0.83	1.25	0.96	0.32									
12	1.72*	2.22*	1.99*	0.98	1.13	1.02	1.02	1.44	1.15	0.51	0.18								
13	2.07*	2.57*	2.34*	1.33	1.48	1.37	1.37	1.79*	1.50	0.86	0.53	0.34							
14	2.21*	2.71*	2.48*	1.47	1.62	1.50	1.51	1.93*	1.64	0.99	0.67	0.48	0.13						
15	2.45*	2.96*	2.72*	1.71*	1.86*	1.75*	1.75*	2.17*	1.88*	1.24	0.92	0.73	0.38	0.24					
16	2.24*	2.74*	2.51*	1.50	1.65*	1.54	1.54	1.96*	1.67*	1.03	0.71	0.52	0.17	0.03	-0.21				
17	2.29*	2.79*	2.56*	1.55	1.70*	1.58	1.59	2.01*	1.72*	1.07	0.75	0.56	0.21	0.07	-0.16	0.04			
18	2.24*	2.74*	2.51*	1.50	1.65*	1.53	1.53	1.95*	1.67*	1.02	0.70	0.51	0.16	0.02	-0.21	0.00	-0.05		
19	2.99*	3.49*	3.26*	2.25*	2.40*	2.28*	2.29*	2.71*	2.42*	1.78*	1.45	1.26	0.91	0.78	0.53	0.74	0.70	0.75	
20	3.56*	4.06*	3.83*	2.82*	2.97*	2.85*	2.86*	3.28*	2.99*	2.34*	2.02*	1.83*	1.48	1.34	1.10	1.31	1.27	1.32	0.56

*Indicates a significant level smaller than 0.05.

 © 2025. The Author(s).
